# Coagulation factor XII deficiency alleviates vascular dysfunction and cognitive impairment in a mouse model of cerebral β-amyloidosis and cerebral amyloid angiopathy

**DOI:** 10.1016/j.nbas.2026.100167

**Published:** 2026-08-25

**Authors:** Marie Rouault, Diana Rita Kindler, Ruiqing Ni, Luca Liberale, Raoul Seiler, Giovanna D. Ielacqua, Roger Nitsch, Christopher Pryce, Giovanni G. Camici, Luka Kulic, Jan Klohs

**Affiliations:** a Institute for Biomedical Engineering, University of Zurich and ETH, Zurich, Zurich, Switzerland; b Zurich Neuroscience Center (ZNZ), Zurich, Switzerland; c AOM-IRCCS Ospedale Policlinico San Martino, Genoa, Italy; d Department of Internal Medicine, University of Genoa, Genoa, Italy; e Department of Animal Phenotyping, Max-Delbrück-Centrum für Molekulare Medizin, Berlin, Germany; f Institute for Regenerative Medicine, University of Zurich, Schlieren, Switzerland; g Preclinical Laboratory for Translational Research into Affective Disorders, University of Zurich, Zurich, Switzerland; h Center for Molecular Cardiology, University of Zurich, Schlieren, Switzerland; i Department of Research and Education, University Hospital Zurich, Rämistrasse 100, 8092 Zurich, Switzerland

**Keywords:** Alzheimer's disease, Coagulation, Hageman factor, Vascular dysfunction, Cerebral microbleeds, Blood-brain barrier, Thrombosis

## Abstract

Amyloid-β (Aβ) can activate the factor XII (FXII)-driven contact system, which exerts several downstream effects on Alzheimer's disease (AD) pathology associated with cognitive impairment. Here, using genetically modified FXII deficient mice crossed with arcAβ mice, we show that genetic deletion of FXII ameliorates β-amyloidosis-mediated susceptibility to arterial thrombus formation, blood-brain barrier leakage, and cerebral microbleed load. Furthermore, we show that genetic deletion of FXII improves cognitive deficits, without affecting Aβ deposition. Thus, the FXII-driven contact system constitutes an important pathway contributing to vascular dysfunction and cognitive impairment in AD, independent of Aβ neuropathology, with important implications for the diagnosis and treatment of the disease.

## 1 Introduction

Alzheimer's disease (AD) is characterized by the deposition of misfolded amyloid-β (Aβ) and tau proteins, leading to neurodegeneration and cognitive impairment [1]. In addition to neuropathology, clinical and experimental studies have demonstrated vascular dysfunction in AD. This dysfunction is characterized by impaired regulation of cerebral blood flow, alterations in vascular morphology and density, impairment of blood-brain barrier (BBB) integrity, and reduced Aβ clearance. Furthermore, there is a strong overlap between AD and cerebral amyloid angiopathy (CAA), which can aggravate vascular dysfunction [2] and predispose patients with AD and cerebrovascular comorbidities to vascular complications.

Emerging evidence has demonstrated abnormalities of the coagulation system in patients with AD and in animal models of the disease. Coagulation factor XII (FXII), also known as Hageman factor, has been shown to be activated in plasma from patients with AD as well as in transgenic mouse models of cerebral β-amyloidosis, indicating activation of the FXII-driven contact system [3], [4]. FXII can be activated by Aβ [4], [5], [6]. Downstream mediators of the FXII-driven contact system can exert multiple effects that are relevant to AD pathology.

Activation of FXII to FXIIa initiates the contact, or intrinsic, coagulation cascade. It triggers the plasma kallikrein-kinin system through cleavage of high-molecular-weight kininogen (HMWK) by prekallikrein to release bradykinin [7]. Increased kallikrein activity, HMWK cleavage [4], and bradykinin levels [8] have been observed in patients with AD. Similarly, decreased levels of FXII, prekallikrein, and HMWK have been observed in various mouse models of cerebral β-amyloidosis [3], [4]. Furthermore, injection of Aβ42 peptide into wild-type mice decreased HMWK levels and increased kallikrein activity [4]. Bradykinin release and activation of its receptors contribute to hemodynamic changes [9], [10] and BBB impairment and stimulate the release of inflammatory cytokines and prostaglandins [10], [11].

In addition, activated FXII initiates the coagulation cascade through activation of FXI, although this process also depends on the tissue factor. Evidence for a prothrombotic state in patients with AD includes increased levels of activated FVII [12], [13], von Willebrand factor [12], [13], prothrombin fragments 1 and 2 [13], fibrinogen α, β, and γ chains [9], and fibrinogen degradation products [13]. Elevated plasma fibrinogen levels have been associated with cognitive decline early in the disease course [14], an increased risk of AD [15], and the degree of brain atrophy [16]. Experimental work, including our own, has shown that fibrin, after cleavage from fibrinogen by thrombin, forms complexes with Aβ that are deposited in cerebral microvessels [17], [18]. These fibrin-Aβ clots are resistant to fibrinolysis and lead to stenosis and loss of vessel perfusion [17]. Moreover, increased fibrin deposition has been observed in post-mortem study of AD patients and found to be associated with synapses loss and neurodegeneration [19].

When BBB function is impaired and downstream mediators of the FXII-driven contact system reach the brain parenchyma, they can elicit an inflammatory response, which may initiate a vicious cycle between coagulation and inflammation [7], [20], [21]. Thus, FXII represents a nexus between coagulation, neuroinflammation, and neurodegeneration in AD and has been identified as a potential therapeutic target. Pharmacological intervention targeting FXII in a mouse model of cerebral β-amyloidosis has been shown to ameliorate neuroinflammation and cognitive impairment [3]. However, the effects of FXII deletion on vascular dysfunction have not yet been studied. To address this question, we investigated susceptibility to thrombosis, vascular dysfunction, Aβ deposition, and cognitive behaviour in a transgenic mouse model of cerebral β-amyloidosis [22] with FXII deficiency.

## 2 Materials and methods

A detailed description of the materials and methods can be found in the **supplementary material.**

### 2.1 Animals

We investigated four experimental groups. Transgenic arcAβ mice, which overexpress the human APP695 with the Swedish and the Arctic (E693G) mutations [22]. The arcAβ strain develops severe CAA with morphological alterations of blood vessels [23], decreased vessel density [18], [24], hypoperfusion [25], [26], impaired vascular reactivity [25], BBB damage [23], [27], [28], and cerebral microbleeds (CMB) [28], [29]. FXII^−/−^ mice, which have normal hemostatic capacity but are protected from pathological thrombosis [30], [31], [32]. ArcAβ mice cross-bred with FXII^−/−^ mice (arcAβ/FXII^−/−^) and non-transgenic littermates (NTL). All animals were around 15 months of age. Animals of both sexes were studied. All experiments were performed in compliance with the Swiss Federal Act on Animal Protection and were approved by the Cantonal Veterinary Office of Zurich (permit 090/16).

### 2.2 Behaviour

The Rotarod test, open field, and Barnes maze were performed in that order. The Rotarod test was performed to evaluate motor activity and motor coordination. The open field test was used to assess spontaneous locomotor activity and exploratory behaviour. The Barnes maze was used to study hippocampus-dependent spatial learning and memory formation, according to published procedures [33], [34].

### 2.3 In vivo arterial thrombosis model

Male mice underwent photochemical injury of the common carotid artery as previously described [35]. The time to vessel occlusion was recorded; blood flow and heart rate were also monitored.

### 2.4 Magnetic resonance imaging

Magnetic resonance imaging was carried out as described [28]. CMBs were counted manually on susceptibility-weighted images.

### 2.5 Trypan Blue BBB extravasation assay

Trypan blue (TB) BBB extravasation assay was performed as previously described [23]. In brief, mice were injected intravenously 200 μL of 0.4% TB solution in 0.85% saline (Gibco, USA). Thirty minutes after injection, mice were transcardially perfused for histological analysis.

### 2.6 Coagulation assays

Blood was collected with cardiac puncture (500 ul) into EDTA-coated tubes (Sarsted, Nümbrecht, Germany) and centrifuged at 1000*g* for 15 min at 4 °C. A mouse FXII sandwich enzyme-linked immunosorbent assay (ELISA) was used for FXII plasma levels analysis. For determination of activated Partial Thromboplastin Time (aPTT) and fibrinogen plasma levels, blood was collected from the right cardiac ventricle with 100 Sterican® 23 G needles (B. Braun, 4657640) and sampled into 3.2% (1:10) sodium citrate micro tubes 0.5 mL 9NC (Sarstedt, Nümbrecht, Germany). Analysis was conducted by the Clinical Laboratory of the Vetsuisse Faculty (University of Zurich, Switzerland) using a Stago Start 4 analyser (Stago Diagnostica, Asnières sur Seine, France). Tissue factor (TF) activity in lysed right carotid arteries was determined by colorimetric ACTICHROME® TF assay as described previously [36].

### 2.7 Histological analysis/Immunohistochemistry

Histological analyses were conducted following published procedures [22], [33] using either immunofluorescence (IF)-staining with 4′,6-Diamidin-2-phenylindol (DAPI) as counterstaining or 3,3-diaminobenzidine (DAB)-staining. Antibodies against amyloid-β plaques (Aβ), endothelial marker (CD31), microglia (Iba1) and astrocytes (GFAP) were used. Thioflavin S staining was used for amyloid-β plaques detection [37].

### 2.8 Microscopy and image analysis

Thioflavin S (IF), Iba1 (DAB) and GFAP (DAB) immunohistochemical images were visualized with a Zeiss Axio Scan.Z1 slidescanner (Carl Zeiss Microspocy, Iena, Germany) and analysed using ImageJ software (NIH, USA). High-resolution and co-localization images of TB, Aβ and CD31 (IF) were acquired using a Leica TCS SP8 (Leica microsystem, Wetzlar, Germany) confocal microscope. CD31 (IF) images were taken with a Leica DM4000B microscope (Leica microsystem, Heerbrugg, Switzerland) using an Olympus DP71 (Olympus, Shinjuku, Tokyo, Japan) colour digital camera and newCast software (Visiopharm, Hoersholm, Denmark). Microscopy images were analysed using ImageJ software threshold segmentation and area measurement tools, and Ilastik (V1.3.2, European Molecular Biology Laboratory, Heidelberg, Germany).

### 2.9 Statistics

Statistical analysis was performed using GraphPad Prism software (V7.00, San Diego, CA, USA). Normality and variance homogeneity was tested with Kolmogorov–Smirnov test and variance homogeneity was assessed with Fisher test for comparison between two groups and Bartlett's test for comparison between more than two groups. As several data sets did not meet the requirement of normality distribution and homoscedasticity, or presented small sample size, statistical analyses were conducted using non-parametric tests as a matter of consistency, except for the statistical analysis of the latency to success in the Barnes maze. For comparison between two groups, Wilcoxon test was used. For comparison between several groups, Kruskal-Wallis test followed by post hoc Dunn's correction for multiple comparison was applied. The latency to success in the learning phase of the Barnes maze was analysed by 4 × 4 (genotype × days) two-way analysis of variance (ANOVA) with repeated measures (RM). Tukey's post hoc test correction for multiple comparison was performed following ANOVA for analysis of the simple effects. A *p*-value <0.05 was considered as statistically significant. In order to go beyond the difference determined by statistical tests and give quantitative information about the difference between two groups when statistical significance was reached and when the assumption of normality was reasonable, we calculated the effect size using Cohen's d [38]. We interpreted the intensity of Cohen's d effect size according to the scale published by Sawilowsky [39] and our knowledge of the variable assessed.

## 3 Results

### 3.1 Higher susceptibility to arterial thrombosis formation in arcAβ mice is rescued by FXII gene deletion

**Fig. 1:**
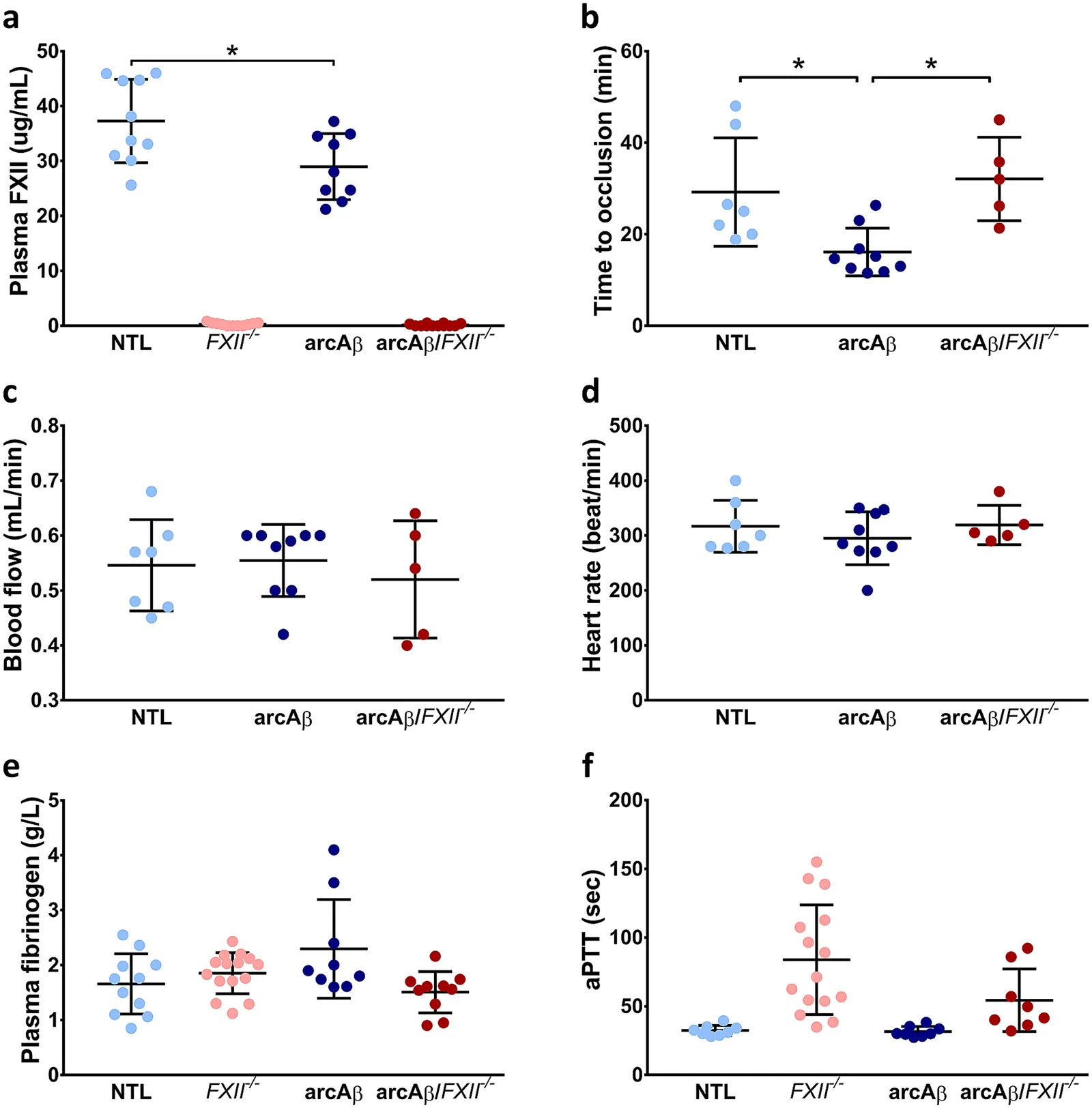
Susceptibility to thrombus formation is increased in the presence of Aβ and mitigated by genetic deletion of FXII. (a) Plasma FXII levels of arcAβ mice were significantly lower in comparison with NTLs. Data presented as mean ± SD. NTL (*n* = 10), FXII^−/−^ (*n* = 11), arcAβ (*n* = 9) and arcAβ/FXII^−/−^ (*n* = 12); Wilcoxon test, *p < 0.05. The deficiency of FXII was confirmed in FXII^−/−^ and arcAβ/FXII^−/−^ mice, which were not considered in the statistical analysis. (b) In vivo photothrombosis in the carotid artery revealed shorter time to occlusion in arcAβ mice compared to NTL and arcAβ/FXII^−/−^ mice. Data presented as mean ± SD. NTL (*n* = 7), arcAβ (*n* = 9) and arcAβ/FXII^−/−^ (*n* = 5); Kruskal-Wallis test followed by post hoc Dunn's correction for multiple comparison, *p < 0.05. (c) Blood flow and (d) Heart rate was monitored during the photothrombosis experiment and did not differ among groups. Data presented as mean ± SD. NTL (*n* = 7), arcAβ (*n* = 9) and arcAβ/FXII^−/−^ (*n* = 5). Kruskal-Wallis test. (e) Plasma fibrinogen levels are not significantly different between NTL, FXII^−/−^, arcAβ and arcAβ/FXII^−/−^ groups. Data presented as mean ± SD. NTL (*n* = 11), FXII^−/−^ (*n* = 15), arcAβ (*n* = 9) and arcAβ/FXII^−/−^ (*n* = 10); Kruskal-Wallis test. (f) NTL and arcAβ mice presented normal aPTT, which were not different between the two groups. As expected, aPTT was prolonged in FXII^−/−^ and arcAβ/FXII^−/−^ mice. Data presented as mean ± SD. NTL (*n* = 8), FXII^−/−^ (*n* = 15), arcAβ (*n* = 8) and arcAβ/FXII^−/−^ (*n* = 8).

We first measured FXII levels in plasma of all groups (Fig. 1a). We confirmed the absence of plasma FXII in FXII^−/−^ mice as previously described [31]. Moreover, we verified that the FXII deficiency phenotype is also preserved in the generated arcAβ/FXII^−/−^ mice. Interestingly, we found lower plasma FXII levels in arcAβ mice compared with NTL (29.0 ± 6.0 vs. 37.3 ± 7.6 μg/mL, *p* = 0.03), suggesting that FXII is consumed in the presence of Aβ and the FXII-driven contact system is activated [3].

We next tested the importance of the Aβ-initiation of the FXII-driven intrinsic pathway of coagulation on arterial thrombosis in vivo (Fig. 1b). The time to occlusion was significantly shorter in arcAβ compared to NTL and arcAβ/FXII^−/−^ (16.1 ± 5.2 vs. 29.2 ± 11.8 and 32.1 ± 9.1 min, *p* = 0.0397 and *p* = 0.0154, respectively), indicating that arcAβ mice are more prone to form an arterial thrombus than NTL and arcAβ/FXII^−/−^ mice. Genetic ablation of FXII in the presence of Aβ mitigated this effect, as the time to occlusion in arcAβ/FXII^−/−^ was not different from NTL mice. Blood flow (Fig. 1c) and heart rate (Fig. 1d) were similar among all experimental groups. As arterial thrombus formation requires also tissue factor, we measured tissue factor activity from the carotid artery but observed no differences between groups (**Supplementary Fig. 1**).

We further performed coagulation assays to assess if activation of the FXII-driven contact system leads to a coagulopathy. Plasma fibrinogen levels were not significantly different between groups (Fig. 1**e**, 1.7 ± 0.5 vs. 1.9 ± 0.4 vs. 2.3 ± 0.9 vs. 1.5 ± 0.4 g/L for NTL, FXII^−/−^, arcAβ and arcAβ/FXII^−/−^ groups, respectively). The aPTT was similar between NTL and arcAβ mice and prolonged in FXII^−/−^ and arcAβ/FXII^−/−^ mice as previously described [31], [40] (Fig. 1f, 32.4 ± 3.7 and 31.5 ± 3.8 vs. 83.9 ± 40.0 and 54.3 ± 22.8 s).

### 3.2 Genetic deletion of FXII alleviates BBB impairment in arcAβ/FXII^−/−^

**Fig. 2:**
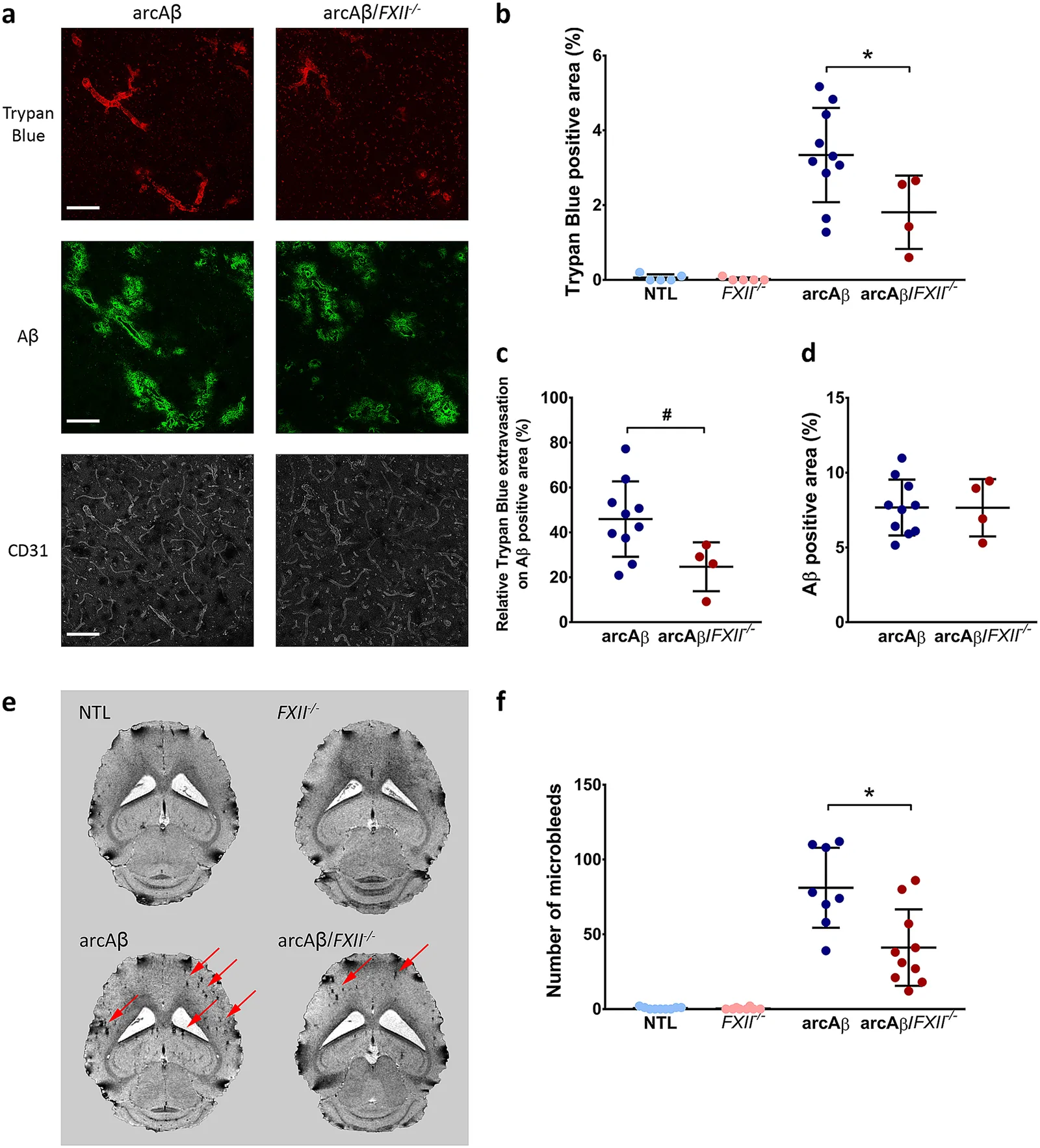
Genetic deletion of FXII ameliorates Aβ-induced BBB impairment. (a) Representative images of TB (red) extravasation in arcAβ and arcAβ/FXII^−/−^ mice. In both groups, TB extravasation was principally observed around middle-size CAA-affected vessels but not around capillaries. Scale bar = 100 μm. (b) TB (red) extravasation was quantified in all groups in relation to Aβ deposition (green). Leakage of TB was evident in arcAβ mice and found blunted by genetic deletion of FXII. Data presented as mean ± SD. NTL (*n* = 5), FXII^−/−^ (*n* = 5), arcAβ (*n* = 10) and arcAβ/FXII^−/−^ (*n* = 4); Wilcoxon test, *p < 0.05. NTL and FXII^−/−^ groups were not included in the statistical analysis in the light of an obvious lack of TB extravasation. (c) When related to Aβ neuropathology, the degree of TB leakage tended to be reduced in arcAβ/FXII^−/−^ in comparison with arcAβ mice, while (d) the overall degree of Aβ deposition was similar between both groups. Data presented as mean ± SD. arcAβ (*n* = 10) and arcAβ/FXII^−/−^ (*n* = 4); Wilcoxon test, *p* = 0.053. (e) CMBs were evident as round/ovoid signal voids in the arcAβ and arcAβ/FXII^−/−^ groups (red arrows). (f) Quantification showed a reduced number of CMBs in arcAβ/FXII^−/−^ mice in comparison with arcAβ mice. Data presented as mean ± SD. NTL (*n* = 9), FXII^−/−^ (*n* = 8), arcAβ (*n* = 8) and arcAβ/FXII^−/−^ (*n* = 10); Wilcoxon test, *p < 0.05. Note that NTL and FXII^−/−^ were not considered in statistical analysis because of the de facto absence of CMBs. (For interpretation of the references to colour in this figure legend, the reader is referred to the web version of this article.)

We next examined the effect of genetic FXII deletion on Aβ-induced BBB impairment. TB extravasation was absent in brain sections of NTL and FXII^−/−^ mice (results not shown). Confirming previously published results in the arcAβ mouse [23], extravasation was mainly observed as subtle leakage around middle-sized vessels in the cortex, presumably cortical penetrating arterioles (Fig. 2a). Capillaries did not seem to present apparent TB leakage. As shown on the microscopy pictures, extravasation was found most of the time in proximity of Aβ plaques, in CAA-affected vessels, and seemed to accumulate within the vessel wall. Compared to arcAβ mice, TB extravasation was attenuated by 45% in arcAβ/FXII^−/−^ mice (3.3 ± 1.3 vs. 1.8 ± 1.0% TB area, *p* = 0.036) (Fig. 2b). As Aβ plaques are believed to play a role in BBB impairment and higher TB leakage may have been due to higher plaque load, we quantified the degree of TB leakage in relation to the degree of plaque deposition. TB extravasation relative to Aβ deposition tended to be alleviated by FXII deletion in arcAβ mice (45.9 ± 16.8 vs. 24.7 ± 10.9% TB/Aβ, *p* = 0.0539) (Fig. 2c), and both groups showed same extent of plaque deposition (Fig. 2d).

### 3.3 Reduction of CMB load in arcAβ/FXII^−/−^ mice

To assess the safety of anti-FXII targeted intervention, we monitored CMB load in all experimental groups. CMBs were very prominent in arcAβ mice (Fig. 2e) but were also observed in arcAβ/FXII^−/−^ mice. In the NTL and FXII^−/−^ groups, only one or two suspected CMBs were occasionally observed. We then counted individual CMBs in animals of all groups. CMB load was reduced by half in arcAβ/FXII^−/−^ mice compared to arcAβ mice (Fig. 2f**;** 41 ± 26 vs. 81 ± 27, *p* = 0.012; Cohen's d effect size result = 1.5). We further assessed differences in the vascular phenotype between groups. Using CD31 IF staining we observed no differences in vessel density (**Supplementary Fig. 2**).

### 3.4 FXII genetic deletion does not alter neuroinflammation in arcAβ/FXII^−/−^ mice

**Fig. 3:**
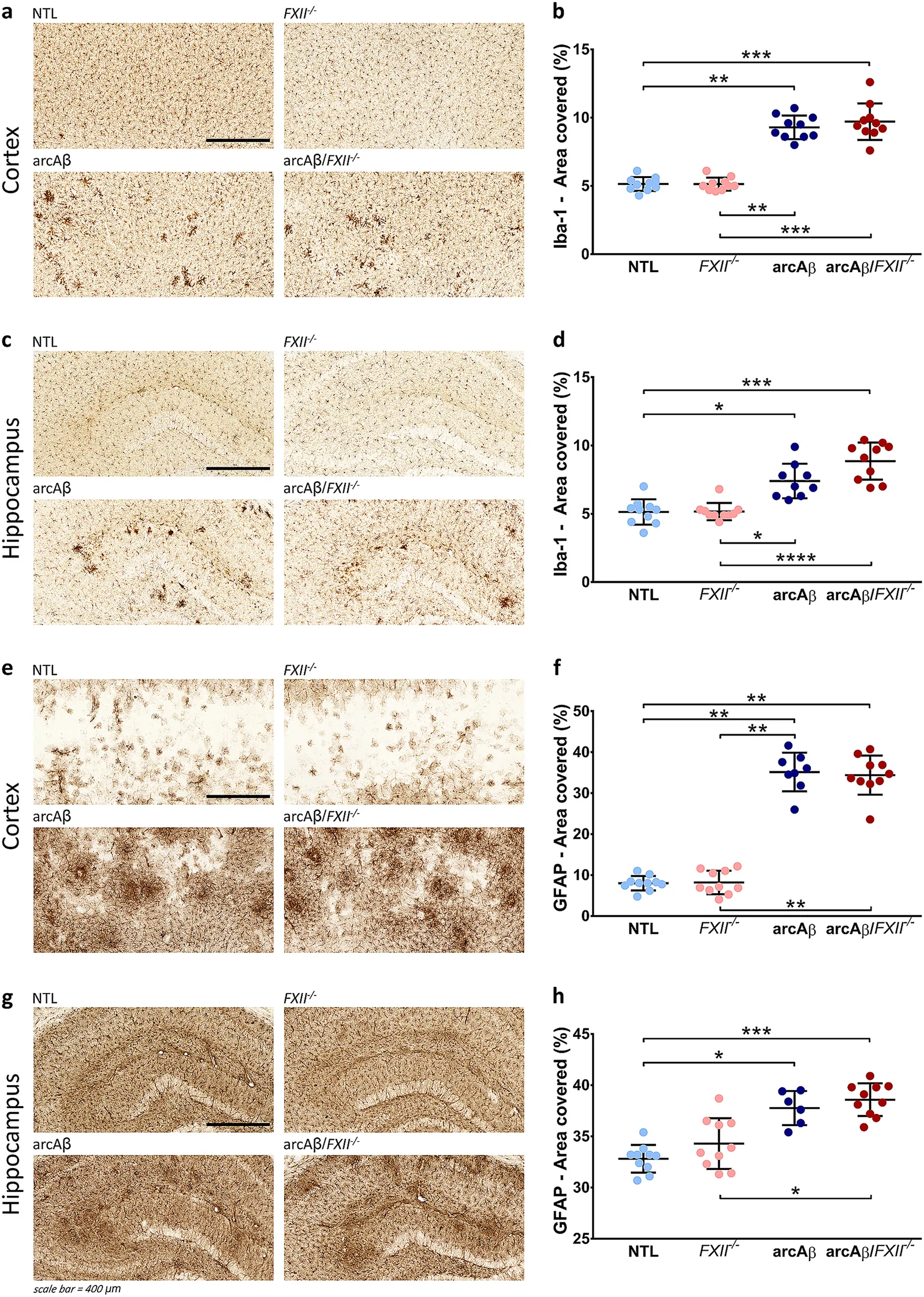
FXII deficiency does not alter Aβ-induced markers of glia activation. (a, c) Immunohistochemical staining of representative brain tissue sections of NTL, FXII^−/−^, arcAβ and arcAβ/FXII^−/−^ groups showing Iba-1 immunoreactivity in the (a) cortex and (c) hippocampus. Quantification of (b) cortical and (d) hippocampal Iba-1 reactivity showed similar levels of microglia activity in arcAβ and arcAβ/FXII^−/−^ mice. (e, g) GFAP immunoreactivity in the (e) cortex and (g) hippocampus. Quantification showed similar levels of astrogliosis in the (f) cortex and (h) hippocampus in arcAβ and arcAβ/FXII^−/−^ mice. All data presented as mean ± SD. NTL (*n* = 10), FXII^−/−^ (*n* = 10), arcAβ (*n* = 6–10) and arcAβ/FXII^−/−^ (*n* = 10). Kruskal-Wallis test followed by post hoc Dunn's correction for multiple comparison, *p < 0.05, **p < 0.01, ***p < 0.001. Scale bar = 400 μm.

Several downstream mediators of the FXII-driven contact pathway of coagulation are pro-inflammatory and have been shown to elicit neuroinflammation when reaching the brain parenchyma [41], [42]. Thus, we investigated the effect of FXII deletion in arcAβ mice on the neuroinflammatory response. Quantitative Iba-1 and GFAP immunohistochemistry was performed for detection of microglia and astrocytes respectively (Fig. 3). ArcAβ and arcAβ/FXII^−/−^ mice exhibited microgliosis and astrocytosis (Fig. 3a,c,e,g bottom row panels). In NTL and FXII^−/−^ mice, no increased microgliosis and astrogliosis were observed (Fig. 3a,c,e,g top row panels). ArcAβ and arcAβ/FXII^−/−^ showed increased Iba-1 expression levels compared to NTL and FXII^−/−^ mice in the cortex (Fig. 3b; 9.3 ± 0.9 vs. 9.7 ± 1.3 vs. 5.1 ± 0.5 vs. 5.1 ± 0.5%) and in the hippocampus (Fig. 3d; 7.4 ± 1.3 vs. 8.9 ± 1.4 vs. 5.1 ± 0.9 vs. 5.2 ± 0.6%). No difference in Iba-1 immunoreactivity were measured between arcAβ and arcAβ/FXII^−/−^ mice. Similarly, GFAP immunoreactivity were higher in arcAβ and arcAβ/FXII^−/−^ compared to NTL and FXII^−/−^ mice in the cortex (Fig. 3f; 35.1 ± 4.7 vs. 34.4 ± 4.8 vs. 8.0 ± 1.8 vs. 8.2 ± 2.3%) and hippocampus (Fig. 3h; 37.8 ± 1.7 vs. 38.6 ± 1.6 vs. 32.8 ± 1.4 vs. 34.3 ± 2.5%). ArcAβ and arcAβ/FXII^−/−^ mice exhibited similar cortical and hippocampal GFAP immunoreactivity. For both immunohistochemical stainings, immunoreactivity was similar between NTL and FXII^−/−^ mice.

### 3.5 FXII deficiency does not alter Aβ deposition in arcAβ/FXII^−/−^ mice

**Fig. 4:**
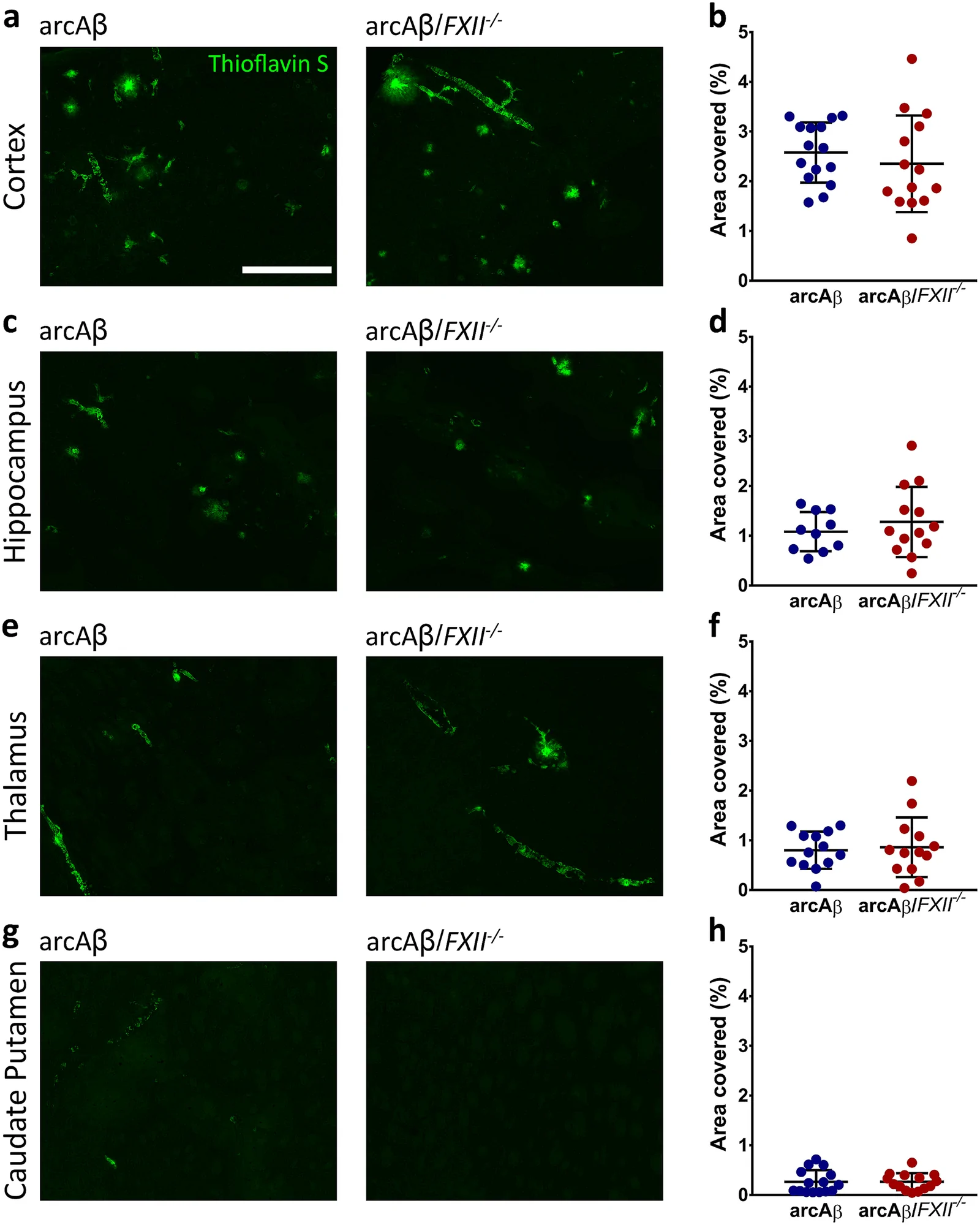
Regional Aβ neuropathology is not affected by FXII deletion. Thioflavin S staining of representative brain sections of arcAβ and arcAβ/FXII^−/−^ mice in the (a) cortex, (c) hippocampus, (e) thalamus, and (g) caudate putamen. Scale bar = 300 μm. (b, d, f, h) Quantification of Thioflavin S staining demonstrated regional differences in Aβ plaques deposition, where the (b) cortex displayed high plaque coverage, the (d) hippocampus and (f) thalamus had moderate and the (h) caudate putamen showed minimal plaques deposition. No difference in plaque load was seen between arcAβ and arcAβ/FXII^−/−^ in the different brain regions. Data presented as mean ± SD. arcAβ (*n* = 10–15) and arcAβ/FXII^−/−^ (*n* = 13–14); Wilcoxon test. Further Thioflavin S quantification in other cerebral areas can be found in Supplementary Fig. 3.

Bradykinin signaling has been shown to regulate the secretory processing of the amyloid precursor protein [43]. Thus, we next asked if genetic deletion of FXII leads to a reduction in cerebral Aβ deposition. We quantified Aβ plaque deposition in arcAβ and arcAβ/FXII^−/−^ mice (Fig. 4**, Supplementary Fig. 3**). We noted regional differences in Aβ plaque deposition, where the cortex showed the highest degree of plaque deposition (Fig. 4a), the hippocampus and thalamus had a milder Aβ deposition (Fig. 4c,e) and some region such as the caudate putamen and hypothalamus were almost exempt of Aβ plaques (Fig. 4g**, Supplementary Fig. 3a**). We did not detect differences between the groups in Aβ load in the cortex (Fig. 4b; 2.6 ± 0.6 vs. 2.4 ± 1.0%), hippocampus (Fig. 4d; 1.1 ± 0.4 vs. 1.3 ± 0.7%), thalamus (Fig. 4f; 0.8 ± 0.4 vs. 0.9 ± 0.6%) and caudate putamen (Fig. 4h, 0.3 ± 0.2 vs. 0.3 ± 0.2%).

### 3.6 FXII genetic deletion improved cognitive performance in arcAβ mice

**Fig. 5:**
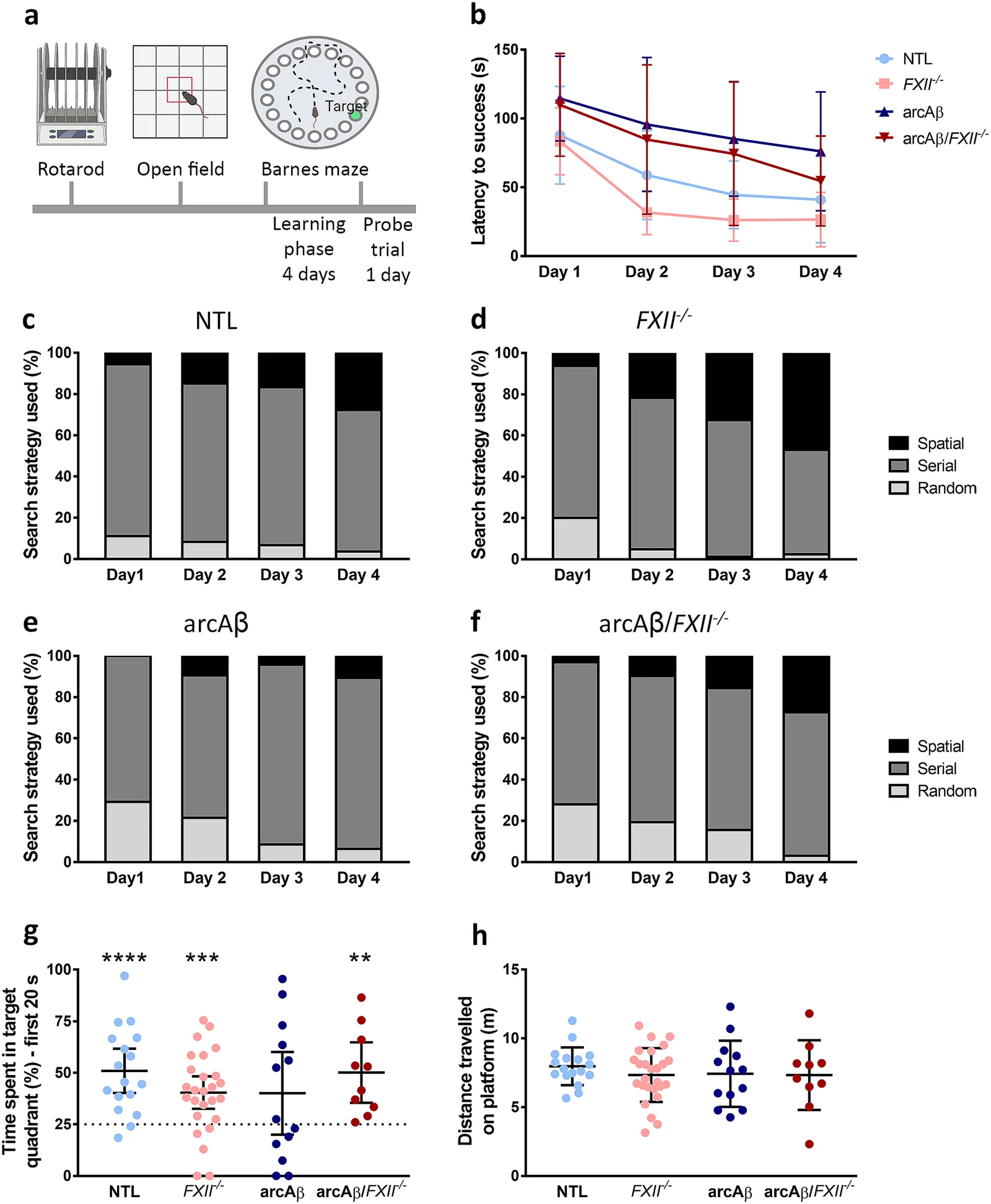
FXII deficiency improved hippocampal-dependent spatial cognition in arcAβ mice. (a) Order of the behaviour tests conducted: Rotarod, followed by Open field and Barnes maze. (b) In the learning phase, the latency to success was determined as the latency until entering the target box. All four groups learned to locate the target box, but the NTL and FXII^−/−^ groups learnt faster than the arcAβ and arcAβ/FXII^−/−^ groups. A graph showing the results of all trials is available in Supplementary Fig. 4a. Data presented as mean of four trials per day ± SD. NTL (*n* = 17), FXII^−/−^ (*n* = 26), arcAβ (*n* = 13) and arcAβ/FXII^−/−^ (*n* = 10); Two-way ANOVA with repeated measures (RM) followed by Tukey's post hoc correction for multiple comparison for analysis of the simple effects. (c, d, e, f) The analysis of the search strategy used in the learning phase showed that NTL, FXII^−/−^ and arcAβ/FXII^−/−^ mice developed more spatial strategy to solve the Barnes maze task compared to arcAβ mice. (g) On the first 20 s of the probe trial, the time spent in the target quadrant was recorded and tested for statistical significance with chance level (25%). The NTL, FXII^−/−^ and arcAβ/FXII^−/−^ groups spent significantly more than 25% of time in the target quadrant but not the arcAβ group. Data presented as mean ± SD. NTL (*n* = 17), FXII^−/−^ (*n* = 26), arcAβ (*n* = 13) and arcAβ/FXII^−/−^ (*n* = 10); Wilcoxon test for comparison of the median with the hypothetical value of 25%, **p < 0.01, ***p < 0.001, ****p < 0.0001. (h) The total distance travelled on the probe trial showed no difference in locomotion among groups. Data presented as mean ± SD. NTL (*n* = 17), FXII^−/−^ (*n* = 26), arcAβ (*n* = 13) and arcAβ/FXII^−/−^ (*n* = 10); Kruskal-Wallis test.

Finally, we interrogated if the beneficial effect of FXII genetic deletion in arcAβ mice on cerebrovascular function were accompanied by an amelioration of the Aβ-induced cognitive deficits (Fig. 5a). Therefore, we assessed hippocampal-dependent spatial learning and memory formation in all four groups. During the learning phase, the successful completion of the task was measured as the latency to enter the target box, in four trials on four consecutive days (Fig. 5b **and Supplementary Fig. 4a**). Statistical analyses of the learning curve using RM two-way ANOVA shows that all groups learned to find the target box as shown by a significant simple effect of day (*p* < 0.0001), but that some experimental groups were better than others in performing the task as the simple effect of genotype was significant (*p* < 0.0001). Analysis of the simple effect genotype showed that NTL learned faster than arcAβ (*p* = 0.0012). FXII^−/−^ performed better than arcAβ (*p* < 0.0001) and arcAβ*/*FXII^−/−^ (*p* = 0.0003) and were also the fastest learner. There were no differences in performance between arcAβ and arcAβ*/*FXII^−/−^ mice in latency to success.

Latency to success (i.e. entering the target box) is a widely used measure of spatial learning and learning efficiency. However, its interpretation is not specific to memory because it may be influenced by non-cognitive factors [44], [45], [46]. In contrast, search strategies (spatial, serial or random) provide complementary information regarding the cognitive processes underlying task performance [45]. Qualitative assessment of the search pattern of all trials of the learning phase (**Supplementary Fig. 5**) revealed that NTL, FXII^*−/−*^ and arcAβ*/*FXII^−/−^ mice had a relative increased proportion of spatial search strategy over the days of the test, supporting the formation of allocentric spatial memory which is hippocampal-dependent in these groups [45] (Fig. 5c,d,e,f**,** percentage of spatial search strategies as follow: NTL, day 1: 5.5%, day 2: 15.0%, day 3: 16.6%, day 4: 27.8%; FXII^−/−^, day 1: 6.3%, day 2: 21.7%, day 3: 32.6%, day 4: 47.0%; arcAβ, day 1: 0.0%, day 2: 9.5%, day 3: 4.3%, day 4: 10.6%; arcAβ/FXII^−/−^, day 1: 3.1%, day 2: 9.7%, day 3: 15.6%, day 4: 27.3%). Conversely, the arcAβ group retained the highest proportion of non-spatial strategies (the majority used serial strategy), indicating that they followed more procedural navigation strategies that are not hippocampal-dependent [47]. This suggests hippocampal-dependent memory deficits in arcAβ mice, since hippocampal impairment may disrupt spatial but not procedural memory [47]. The higher proportion of spatial strategy in arcAβ*/*FXII^−/−^ mice suggests that they had a more intact hippocampal-dependent memory compared to arcAβ mice which showed a higher proportion of serial strategy.

During the probe trial, the latency to first entry the escape box was measured but did not reveal statistical differences among groups (**Supplementary Fig. 4b**). Nevertheless, comparing only the two groups NTL and arcAβ in a Wilcoxon test revealed a longer latency of arcAβ compared to NTL. Spatial memory retention was evaluated by recording the time spent in the target quadrant in the first 20 s of the test and comparing it with the 25% chance level, which corresponds to the probability that an animal spent time in one of the quadrants (Fig. 5g). ArcAβ mice presented a deficit in retention memory as shown by the time spent in the target quadrant, which was not significantly different from chance level (25%) (Fig. 5g, 40.1 ± 33.1% time spent in target quadrant, non-significant), in accordance with previous finding in the arcAβ line [22]. Conversely, NTL, FXII^−/−^ and including arcAβ*/*FXII^−/−^ mice spent significantly more time than 25% chance level in the target quadrant (Fig. 5g, 51.1 ± 20.9 vs. 40.5 ± 19.5 vs. 50.1 ± 20.5% time spent in target quadrant, *p* = 0.0001, *p* = 0.0008, *p* = 0.002, respectively). This shows that NTL, FXII^−/−^ and including arcAβ/FXII^−/−^ preferentially searched within the target quadrant, suggesting successful spatial memory retention, while this was not the case for arcAβ mice. We also checked for locomotion in the probe trial by recording the total distance travelled on the platform and did not find differences among groups (Fig. 5h).

**Fig. 6:**
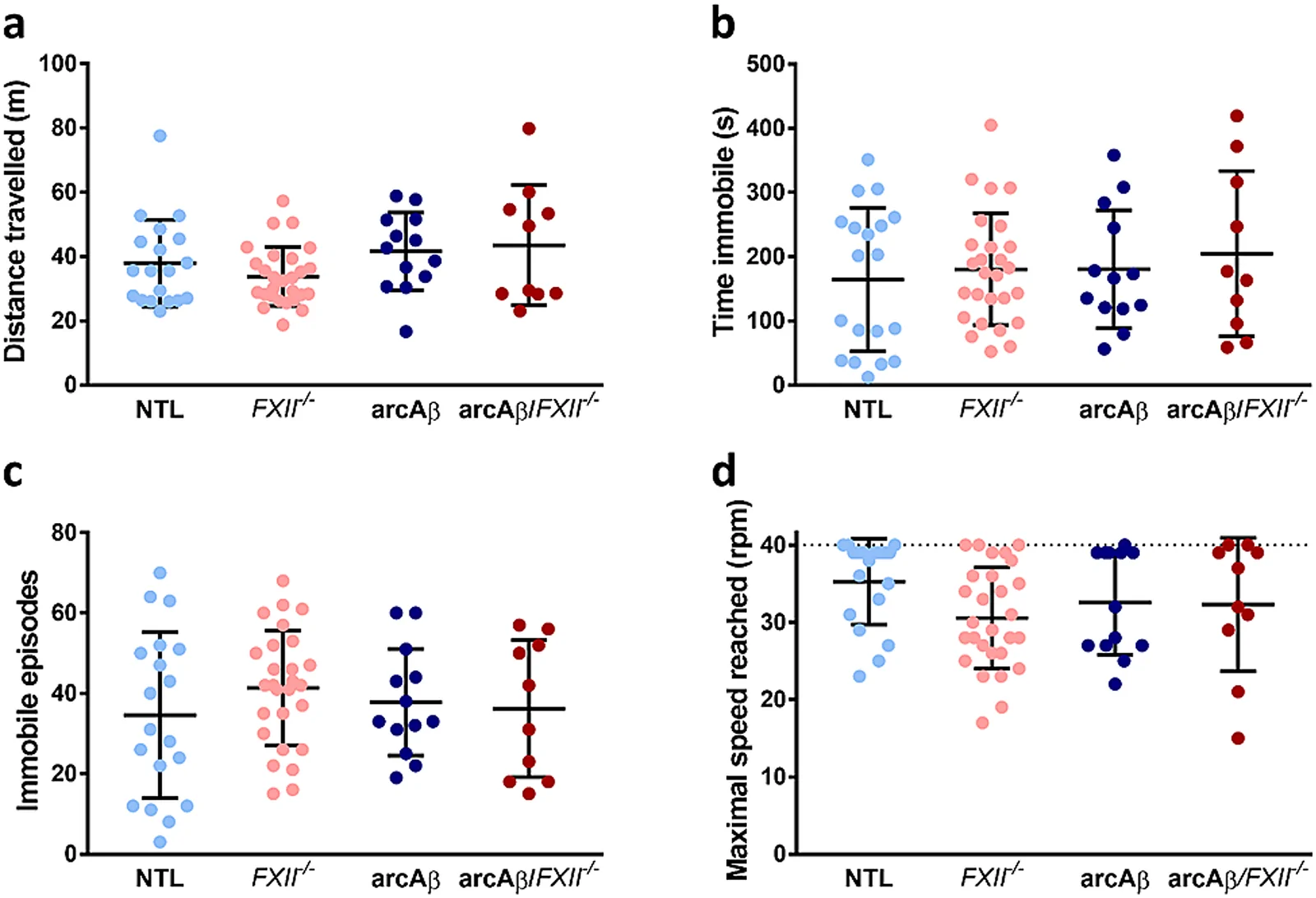
FXII deficiency does not affect basic locomotor functions in NTL and arcAβ mice. (a) Locomotion measured in the open field test was similar among NTL, FXII^−/−^, arcAβ and arcAβ/FXII^−/−^ groups. (b, c) Time immobile and immobile episodes analysed in the open field test were not affected by genotype. Data presented as mean ± SD. NTL (*n* = 19), FXII^−/−^ (*n* = 27), arcAβ (*n* = 13) and arcAβ/FXII^−/−^ (*n* = 10); Kruskal-Wallis test. (d) The Rotarod test for motor coordination revealed no difference in the maximal speed reached among all four groups. Data presented as mean ± SD. NTL (*n* = 19), FXII^−/−^ (*n* = 28), arcAβ (*n* = 13) and arcAβ/FXII^−/−^ (*n* = 10); Kruskal-Wallis test.

As the Barnes maze is a spatial navigation task, and differences in locomotion could induce a bias in the cognitive performance results, but in the open field test all experimental groups presented the same level of locomotor activity and exploration as shown by similar distance travelled in the box, time immobile and number of immobile episodes (Fig. 6a,b,c). In the Rotarod test, no difference in maximal speed reached was measured among the four groups (Fig. 6d).

## 4 Discussion

Our findings show that FXII deficiency mitigates Aβ-mediated vascular and cognitive dysfunction in the arcAβ mouse model of cerebral β-amyloidosis with CAA. Crossing FXII^*−/−*^ with arcAβ mice effectively prohibited activation of the FXII-driven intrinsic coagulation pathway and significantly reduced the susceptibility to arterial thrombus formation, alleviated BBB leakage and reduced CMB load, that collectively constitute important cerebrovascular pathways to cognitive impairment in AD [48]. We found that these changes were not accompanied by improvements of Aβ neuropathology and histological effects on glia.

### 4.1 FXII-driven contact activation contributes to susceptibility to arterial thrombosis in cerebral β-amyloidosis

At 15 months of age, severe cerebral β-amyloidosis and CAA pathology is established in arcAβ mice [22], [23]. ArcAβ mice had lower plasma FXII levels suggesting contact activation of blood-circulating FXII zymogen to the serine protease FXIIa by Aβ [49]. This is in line with previous reports showing regulation of FXII and its downstream mediators in AD patients and animal models of cerebral amyloidosis ([3]; [8]; [4]). The absence of detectable FXII in arcAβ/FXII^−/−^ mice demonstrated that the double mutant can be used to study the effect of FXII deficiency in the presence of cerebral β-amyloidosis and CAA.

Activated FXII initiates the intrinsic coagulation pathway which is critically involved in thrombosis [50]. When photothrombosis in the right common carotid artery was performed, the time until complete vessel occlusion was shorter in arcAβ compared to NTL indicating that arcAβ mice are more prone to arterial thrombus formation. This is in line with previous reports showing accelerated thrombus formation in ferric chloride-induced injury of the right carotid artery in APP23 [51], and TgCRND8 mice [17], which are mouse models that develops CAA at late stage of the disease. In contrast to the ferric-chloride approach, our model of arterial thrombosis is triggered by endothelial-specific damage due to free radicals released by Rose Bengal after laser stimulation and therefore is thought to better represent human pathophysiology. Importantly, FXII deficiency in arcAβ/FXII^−/*−*^ mice led to a normalization of the occlusion time to values similar of NTLs.

The aPPT was prolonged in FXII^−/−^ mice as described [31], [32], [40]*,* confirming the lack of integrity of the coagulation cascade due to the absence of FXII*.* However, both aPTT and plasma fibrinogen levels were similar between NTL and arcAβ mice. Increase fibrinogen levels in AD patients plasma have been detected [6] and have been linked with higher risk of developing dementia [15]. Systemic changes in coagulation properties in arcAβ mice might be too low or too short-lived to be detected with coagulation assays. Nonetheless, we have previously described local fibrinogen deposition in conjunction with Aβ in cerebral blood vessels in the arcAβ mouse [18].

### 4.2 FXII deficiency alleviates BBB impairment without increasing the risk of cerebral bleeding

Activation of the kallikrein-kinin system results in the formation of bradykinin from HMWK through cleavage by kallikrein. Increased plasma bradykinin levels have been measured in AD patients [8] and in the arcAβ mouse model [25]. Bradykinin induces BBB permeability via opening of tight junctions [10], [11]. BBB disruption has been shown as an early important contributor to cognitive impairment [52], [53] and the degree of impairment correlates with disease progression [54]. Thus, we asked the question if deficiency of FXII in arcAβ/FXII^−/−^ mice, upstream of bradykinin, improves BBB permeability present in arcAβ mice [28], [31]. Firstly, we observed that genetic deletion of FXII attenuated TB extravasation by 45% compared to arcAβ mice. Secondly, we assessed CMB load in all groups and found a 50% reduction in CMB load in arcAβ/FXII^−/−^ mice compared to arcAβ mice. Our data indicates that targeting the FXII-driven contact system in the arcAβ mouse model of cerebral β-amyloidosis and CAA markedly alleviates BBB impairment.

### 4.3 Amelioration of cognitive dysfunction occurs independently of Aβ neuropathology and histological effects on glia

Activation of the FXII-driven contact pathway of coagulation has been shown to induce pro-inflammatory responses in the brain in AD and neuroinflammatory disorders [41], [42]. Activation of bradykinin receptors by bradykinin initiates release of inflammatory cytokines (e.g. TNF-α, IL-1β) and prostaglandins [9], [10]. Downstream mediators of the FXII-driven contact system such as thrombin and fibrinogen can reach the brain parenchyma and elicit and activate microglia [17], [19], [42]. Increased levels of inflammatory cytokines and chemokines [55], [56], and reactive microglia [22], [23], [27], [57] are observed in AD patients and mouse models of AD, where inflammation has been implicated to contribute substantially to the disease progression and severity [57]. Using Iba-1 and GFAP immunohistochemistry we found that arcAβ/FXII^−/−^ had similar Iba-1 and GFAP immunoreactivity than arcAβ mice. This is in contrast to an earlier study which showed that FXII depletion in TgCRND8 mice using antisense oligonucleotide-mediated messenger RNA knockdown results in reduced microglia and astrocytes activity [3]. The difference may be explained by differences in the transgenic mouse models used, where microglia and astrocytes occur earlier in the TgCRND8 [58] than in the arcAβ mice [22].

We next assessed the effect of factor XII deletion on cerebral Aβ deposition. It was shown that stimulation of B_2_R by bradykinin regulates the secretory processing of the APP in vitro by stimulating α-secretase and decreasing Aβ production in skin fibroblasts from patients with familial AD [43]. However, animal studies investigating the effect of BRs on Aβ load have revealed contradictory results [41], [59], [60]. We have previously shown that chronic treatment with the B_1_R and B_2_R blocker noscapine in arcAβ mice did not alter cerebral Aβ deposition [25]. Furthermore, FXII depletion using antisense oligonucleotide-mediated messenger RNA knockdown has not led to changes in cerebral Aβ pathology in TgCRND8 mice [3]. In line with this finding, we have seen no effect of genetic FXII deletion in arcAβ mice in any of the brain region investigated. It is likely that targeting FXII-driven contact activation system mainly exerts its beneficial effects by acting on the cerebral vasculature and circulation, and that its contribution to neuropathology is limited.

The downstream mechanisms of FXII activation on brain vascular functions are relevant for cognition and activation of the FXII-driven contact system has been suggested to be associated with cognitive impairment in AD patients and mouse models ([3], [17], [60], [61], [62], [63]; [8]; [64]). Thus, we examined the effects of FXII deletion in arcAβ mice on cognitive performance. In the Barnes maze arcAβ mice showed hippocampal-dependent spatial cognitive deficits, confirming previous characterization of this line [22]. We found that arcAβ/FXII^−/−^ mice had relatively more spatial search strategy in the learning phase than arcAβ mice, and that they performed significantly above chance level in the retention memory trial, but arcAβ did not, implying that FXII deletion had a positive effect on cognition in arcAβ mice. These findings suggest that arcAβ*/*FXII^−/−^ mice had a more intact hippocampal-dependent memory in contrast to arcAβ mice.

### 4.4 Therapeutic implications of targeting FXII in AD and CAA

The beneficial effect on cognition of FXII deficiency in the arcAβ mouse model of cerebral β-amyloidosis and CAA corroborates a previous report showing that depletion of plasma FXII using antisense oligonucleotide-mediated messenger RNA improved cognitive function in the retention memory phase of the Barnes maze in a mouse model of cerebral β-amyloidosis [3]. Likewise, inhibition of other key players of the FXII-driven contact system have been shown to provide cognitive benefits in various AD mouse models [17], [60], [61], [62]. Genetic deletion or pharmacological inhibition of B_1_R or B_2_R improved cognitive impairment in an Aβ-injected mouse model of AD [62]. Antagonism of B_1_R was similarly beneficial for spatial learning and memory in a transgenic APP mouse model [60]. Treatment with the defibrinogenating agent ancrod or a fibrinogen inhibitor ameliorated spatial memory in mouse models of cerebral β-amyloidosis [17], [61].

FXII constitutes a potential therapeutic target in AD, which is expected to be of high clinical relevance. Conventional anticoagulant therapy (warfarin, heparin) have been shown to have beneficial effect on cognition in patients with dementia [65], [66] and in AD mouse models pathology [67] but have been linked to an increased risk of bleeding and intracerebral hemorrhages [68] particularly in patients with CAA [69], [70].

In humans, hereditary deficiency of FXII is not associated with abnormal bleeding [71]. Similarly, FXII^−/−^ mice do not experience spontaneous or excessive injury-related bleedings [31], [32]. In the current study, we show that FXII deletion in the presence of CAA in arcAβ/FXII^−/−^ mice does not increase the risk of cerebral bleedings but even reduces CMB load. Thus, a treatment depleting FXII has the potential to be favorable in AD patients affected by CAA and bypass bleeding risks often associated with conventional anticoagulant therapy. Moreover, FXII-targeted therapy that is currently coming to the market might be easy to administer via the circulatory route [72].

### 4.5 Limitations and future directions

In the current study we used a transgenic mouse model of cerebral β-amyloidosis and CAA with FXII deficiency. The approach was well suited to achieve the absence of plasma FXII in arcAβ/FXII^−/−^ and FXII^−/−^ mice and thus to study downstream effects of the FXII^−/−^ deficiency in the β-amyloid-induced pathology. The approach does not model a potential clinical situation in which FXII^−/−^ activity is suppressed during a specific therapeutic window. Moreover, although FXII-deficient mice are viable and do not show evidence of a major developmental requirement for FXII, constitutive gene deletion may still allow developmental or lifelong compensatory adaptations that would result in differences from an acute therapeutic FXII inhibition. Future studies using temporally controlled genetic or pharmacological approaches such as antisense oligonucleotides [3], RNA interference, FXII knockdown or antibody-based treatments would better mimic therapeutic intervention and help define the optimal treatment window.

Another limitation is that the study was performed in a single transgenic mouse model of cerebral β-amyloidosis and CAA at an advanced disease stage. Although the arcAβ model is well suited to study vascular pathology [18], [22], [23], [24], [25], [26], [27], [28], [29], findings may not fully generalize to other AD models, to earlier disease stages, or to the heterogeneous pathology observed in patients with AD. A previous study has shown in the TgCRND8 model that FXII-targeted intervention improves cognitive function amyloidosis [3], though read-outs of vascular pathology such as BBB impairment were not investigated. Future studies should therefore test FXII-targeted interventions across additional models of cerebral β-amyloidosis at different disease stages. Moreover, it remains to be determined whether FXII-targeted therapy is particularly beneficial in models with prominent CAA, where vascular Aβ is more directly exposed to circulating FXII and may therefore more strongly activate the FXII-driven contact system.

The downstream cellular and molecular pathways that mediate the vascular and cognitive benefits in arcAβ/FXII^−/−^ mice were not investigated in this study in detail. We found that the observed improvements occurred independently of changes in Aβ deposition and without alterations in Iba1/GFAP immunoreactivity. However, we did not investigate other potential mediators of the coagulation, hemodynamic, kallikrein-kinin and neuroinflammatory pathways that are known to exert effects on cognitive function [3], [17], [18], [19]. We have previously shown that fibrin(ogen) deposition and bradykinin signaling in the arcAβ model are linked to hypoperfusion and impaired vascular reactivity [18], [25], both of which contribute to cognitive impairment in patients with AD [48]. Future studies should assess the levels of bradykinin signaling, fibrin(ogen) deposition and microvascular occlusions, thrombin activity, kallikrein activation, HMWK cleavage, and inflammatory cytokine levels in the brain and plasma. Moreover, investigations of neuroinflammation should go beyond the histological assessment of glia and include cytokine/chemokine profiling, microglial activation states, and transcriptomic analyses in the different groups.

Taken together, we have shown that abrogation of the FXII-driven pathway constitutes an effective and safe way to ameliorate BBB impairment and a pro-thrombotic state in an established mouse model of AD. Effects on vascular function was associated with an improvement of the cognitive deficit, independent of Aβ deposition and histological effects on glia. Therapeutic targeting of FXII might therefore represent promising therapeutic strategy to mitigate dysfunction of the cerebral and systemic vasculature in AD and CAA.

## CRediT authorship contribution statement

**Marie Rouault:** Writing – original draft, Methodology, Investigation, Formal analysis, Data curation, Conceptualization. **Diana Rita Kindler:** Writing – review & editing, Methodology, Investigation. **Ruiqing Ni:** Writing – review & editing, Investigation, Formal analysis, Data curation. **Luca Liberale:** Writing – review & editing, Investigation, Formal analysis, Data curation. **Raoul Seiler:** Writing – review & editing, Methodology, Investigation. **Giovanna D. Ielacqua:** Writing – review & editing, Methodology, Investigation, Formal analysis. **Roger Nitsch:** Writing – review & editing, Supervision, Resources. **Christopher Pryce:** Writing – review & editing, Supervision, Formal analysis. **Giovanni G. Camici:** Writing – review & editing, Supervision, Funding acquisition, Formal analysis, Data curation, Conceptualization. **Luka Kulic:** Writing – review & editing, Validation, Supervision, Funding acquisition, Data curation, Conceptualization. **Jan Klohs:** Writing – original draft, Supervision, Resources, Project administration, Methodology, Investigation, Funding acquisition, Formal analysis, Data curation, Conceptualization.

## Ethics declaration

### Animal subject

This study was conducted in accordance with the ARRIVE (Animal Research: Reporting of In Vivo Experiments) guidelines. This study was approved by the Cantonal Veterinary Office of Zurich. (Approval No. 090/16)

## Funding

This work was funded by the University of Zurich and the ETH Zurich Foundation through a Seed Grant of “University Medicine Zurich/Hochschulmedizin Zürich”, Synapsis Stiftung, Olga Mayenfisch Stiftung, Hartmann Müller Stiftung, and Vontobel foundation.

## Declaration of competing interest

The authors declare the following financial interests/personal relationships which may be considered as potential competing interests: Luca Liberale and Giovanni G. Camici are coinventors on the International Patent WO/2020/226993 filed in April 2020. The patent relates to the use of antibodies which specifically bind IL-1α to reduce various sequelae of ischemia-reperfusion injury to the central nervous system. GC is educational consultant to the Zurich heart House Foundation. The other authors declare that they have no financial conflict of interest.
