# Supplementary material for: Coagulation factor XII deficiency alleviates vascular dysfunction and cognitive impairment in a mouse model of cerebral β-amyloidosis and cerebral amyloid angiopathy
Source: Aging Brain. 2026 Aug 25;10:100167. doi: 10.1016/j.nbas.2026.100167 (PMC13543942; doi:10.1016/j.nbas.2026.100167)
Supplement: Supplementary file 1 [file mmc1.docx]

**Supplementary Material**

**Materials and Methods**

**Animals and housing**

ARRIVE guidelines for reporting animal research were followed. Transgenic arctic mice overexpressing the human APP695 with the Swedish and the Arctic (E693G) mutations (arcAβ) (Knobloch et al., 2007), FXII deficient mice (*FXII^-/-^*) (Pauer et al., 2004), a crossbred of the arcAβ and *FXII^-/-^* mouse lines (arcAβ/*FXII^-/-^*) and non-transgenic littermates (NTL) were used in the study. Animals were backcrossed for at least 10 generations on a C57/Bl6 background and mice of both genders were used. Mice were bred at the EPIC Phenomic centre (ETH Zurich, Switzerland). Animals were housed in individually ventilated acrylic cages (194 x 181 x 398 mm) with wood shavings bedding, 12/12-hour (h) light/dark cycle at 21°C, 55-60 % humidity with *ad libitum* access to water and standard chow (3437PXL15, CARGILL). Paper tissue and red Tecniplast mouse house® (Tecniplast, Milan, Italy) shelters were placed in cages as environmental enrichments. To ensure quality control, mice were genotyped from biopsies taken after birth and after sacrifice.

**Behaviour**

**Experimental conditions**

Mice were placed in an inverted light cycle housing room (dark phase from 9:00 am to 9:00 pm) 18 days before the start of the behaviour experiments and during behaviour tests. During the acclimatization period, mice were handled every two to three days and were accustomed to being cupped with open hand to minimize anxiety (Hurst et al., 2010). The mice were also accustomed to stay alone in the test cage, in which the animal was placed before the start of the experiment and during the experiment in case of an intertrial period. The mice were monitored for health and body weight and only mice with suitable health were further allowed in the experiments. One experimenter (female) was involved in the acclimatization and behaviour tasks. The experimenter was blinded to the genotype.

Behaviour tests were performed during the active phase of the mice, i.e. dark phase, and were started one hour after beginning of dark onset. Mice used for behaviour were housed in groups of 2 to 5 animals, except for a few male mice that were single-caged due to aggressive behaviour. Single-caged male mice were all included in behaviour tests. Genotype order was randomized for the behaviour tests. During testing, mice were placed in the behaviour experimental room the night before the test. The experimenter was present in the test room for the entire duration of the test procedure but was out of the test area surrounded by black curtains and thus not visible when the mice were tested. A camera placed above the different apparatus recorded the behaviour data. The ANY-maze software (Stoelting, IL, USA) was employed to track the mice and to analyse the behaviour data.

The order of the test performed was the following: (1) Rotarod, (2) open field, (3) Barnes maze. Between two tests, mice had a recovery period between one to ten days.

**Rotarod**

As the Barnes maze test is based on spatial navigation, which success depends on motor coordination, the Rotarod (Ugo Basile, Varese, Italy) test was first conducted to clarify if mice of any genotype present with a motor deficit (Brooks et al., 2009; Gawel et al. 2019). Mice were placed on a horizontal cylinder rotating under continuous acceleration from 5 round per min (rpm) to 40 rpm within 3 min. Latency to fall (or maximum performance scored 40 rpm or 180 s was recorded. The mice had three sessions on different days. During each session, mice executed the Rotarod test three time and the best performance of the last session was kept for analysis.

**Open field**

The open field test is a simple method used to assess spontaneous locomotor activity and exploratory behaviour (Brooks et al., 2009). The apparatus consisted of a white square box (49 × 49 × 33 cm) divided into a central zone in the middle of the arena under a light of 20 Lux and a peripheral zone with a luminosity of 10 Lux. The mouse was placed in one corner of the box and let free to explore the environment for 15 min. After each animal trial, the box was cleaned with water, 3 % H_2_O_2_ and water. The ANY-maze software (Stoelting, Wood Dale, IL, USA) recorded total distance travelled as a measure of locomotion. One *FXII^-/-^* mouse aged 15 months was excluded from the analysis because of outlier locomotion value (> 100 m).

**Barnes maze**

The Barnes maze was used to study hippocampus-dependent spatial learning and memory formation (Rosenfeld et al., 2014; Harrison et al., 2006), according to published procedures (Kulic et al., 2012; Derungs et al., 2017). The apparatus consisted of a grey circular platform of 91 cm diameter with an even illumination of 40 Lux, elevated 90 cm above the ground, with 20 holes at its circumference. Under one hole was placed a target box, the other being connected to false boxes, which were too shallow for the mouse. The 19 false boxes were not visible from the centre of the maze where the mouse was placed at start. The high illumination, the open area and the abyss provided motivation to search for the escape box. The maze was surrounded by black curtains on which a visual cue was taped in front of the escape box (Pompl et al., 1999).

The training phase consisted of four subsequent days with four trials per day where the mouse was trained to locate the target box and enter it via a hole to successfully escape the maze. Before starting the test, four mice were placed individually in empty cages that also served as intertrial cages. The first mouse was put in the centre of the platform, the head directed opposite to the target box. A non-transparent box was placed over the mouse. The test started when the box was lifted and the mouse was allowed to explore the maze and find the target box. If the mouse located the target hole and entered the target box, the trial was stopped, and the mouse was directly brought back to the intertrial cage. If the mouse did not enter the target box after the three min of the test had elapsed, the mouse was gently guided with hands to enter the target box and was placed back to the intertrial cage. The maze and the target box were then cleaned with 70 % ethanol. The same procedure was repeated with the second, the third and the fourth mouse. When the first trial was completed for all four mice, the first mouse was taken to start the second trial. When all four trials were completed for all four mice, the mice were placed back in their home cage. The intertrial period lasted 4 to 15 min, with an average of 10 min for most of the mice.

The probe trial occurred on the fifth day, 24 h after the training phase. The target box was removed, and a false box was put in place. The animal was allowed to explore the maze for 90 s during one single trial. The ANY-maze software was employed to record and analyse parameters during the learning phase, such as latency to successfully enter the target box (called latency to success), and during the retention memory trial, such as distance travelled on platform, latency to reach the target hole, time spent in target quadrant. Two NTL mice aged 15 months who did not enter the target box (latency to success scored 180 s) at all trials on day 3 and day 4 and two FXII^-/-^ with outlier values were excluded from the analysis.

Search strategy of the learning phase were determined using an automated classification algorithm called Barnes-maze unbiased strategy (BUNS) based on a support vector machine (Illouz et al. 2016). The automated method enables to avoid the bias and potential inconsistency induced by manual classification of the track pattern by an experimenter. The BUNS algorithm examined each track plot generated by the ANY-maze software and classified them into six search strategies: random (the mouse does not show ability to locate the target hole and moves randomly on the platform), serial (the mouse visits several adjacent holes in a serial manner), focused search (the mouse scans the location around the target hole), long correction (the mouse moves to a distal position from the target hole and makes a major correction re-directing its trajectory towards the target hole), corrected (the mouse trajectory does a minor correction toward the target hole), and direct (the mouse moves directly toward the target hole and enter the target box) (Illouz et al., 2016). For a clearer and simpler presentation of the search strategy, we grouped focused search, long correction and serial strategies together in the serial search category, and we grouped the most highly spatial strategy corrected and direct into a category called spatial. Thus, the search strategy presented are classified in three categories: random, serial and spatial, in accordance with the spatial navigation criteria as defined (Harrison et al., 2006).

***In vivo* arterial thrombosis model**

Male mice underwent photochemical injury of the common carotid artery as previously described (Liberale et al., 2001). Briefly, mice were anaesthetized by intraperitoneal injection of 87 mg/kg sodium pentobarbital (Butler, Columbus, OH, USA). The depth of anaesthesia was confirmed by the absence of twitch reflex. Rose Bengal (Fischer Scientific, Fair Lawn, NJ, USA) was diluted to 12 mg/ml in phosphate-buffered saline and then injected through the tail vein at a concentration of 63 mg/kg. Mice were placed in supine position under a dissecting microscope, and the right common carotid artery was exposed by a midline cervical incision. A Doppler flow probe (Model 0.5 VB, Transonic Systems, Ithaca, NY, USA) was applied and connected to a flowmeter (Model T106, Transonic Systems, Ithaca, NY, USA). Five to ten min after Rose Bengal injection, a 1.5 mW green light laser (540 nm; Melles Griot, Carlsbad, CA, USA) was applied to the site of injury 6 cm away from the artery for 60 min or until thrombosis occurred. From the onset of injury, carotid blood flow and heart rate were continuously monitored up to 120 min. Occlusion was defined as blood flow below 0.1 ml/min for at least 1 min.

**Magnetic resonance imaging**

MRI was carried out on a Bruker Biospec 94/30 (Bruker Biospin) small animal MR system operating at 9.4 T (Klohs et al., 2011). The system was equipped with a cryogenic 2 × 2 radiofrequency surface coil probe (Bruker BioSpin). Mice were anesthetized with an initial dose of 4 % isoflurane in oxygen/air mixture (200:800 ml/min) and were maintained at 1.5 % isoflurane. Mice were endotracheally intubated and mechanically ventilated with 90 breaths per minute (bpm), applying a respiration cycle of 25 % inhalation and 75 % exhalation (MRI-1 Volume Ventilator CWI Inc., Ardmmore, PA, USA). Body temperatures were monitored with a rectal temperature probe (MLT 415, AD Instruments, Spechbach, Germany) and kept at 36.5 ± 0.5 °C on a water-heated holder (Bruker Biospin). Anatomical reference data was acquired in coronal and sagittal directions. After standard adjustments, 3D gradient echo was acquired with TE = 12 ms; TR = 250 ms; FA = 15°, no averaging; FOV = 15 × 12 × 2.2 mm; matrix size = 248 × 199 x 36; spatial resolution = 60 × 60 × 61 μm; within a total scan time = 29 min 51s. SW images were computed from gradient echo data using the SWI processing module in ParaVision 6.0.1 (Bruker) as described (Klohs et al. 2011; Ni et al., 2020). Reconstruction was performed with a Gauss broadening = 1 mm and a mask weighting = 4. CMBs were counted manually twice using MRIcron ([www.sph.sc.edu/comd/rorden/mricron/](http://www.sph.sc.edu/comd/rorden/mricron/)) (Klohs et al., 2011). CMB were defined by their isolated circular or ovoid shape across slices and could be distinguish from vascular structures from veins that give also rise to contrast in SW images.

**Trypan Blue BBB extravasation assay**

Trypan blue BBB extravasation assay was performed as previously described (Persson et al., 1976; Merlini et al., 2011). Mice were prepared for tail vein injection under a short anaesthesia with 2% isoflurane (Abbott, Cham, Switzerland) in air/oxygen (200:800 ml/min) mixture. Mice were injected intravenously 200 μl of 0.4% Trypan Blue solution in 0.85% saline (Gibco, USA). Thirty minutes after injection, mice were anesthetized using a cocktail of ketamine/xylaxine/acepromazine (75/10/2 mg/kg b.w.). Mice were transcardially perfused with 20 ml ice-cold PBS followed by 50 ml 4% PFA in PBS. Brains were postfixed in 4% PFA in PBS for 4 h at 4°C for histological analysis.

**Coagulation assays**

Mice were anaesthetized with an overdose of sodium pentobarbital (100 mg/kg) and blood was collected from the right cardiac ventricle with 100 Sterican® 23 G needles (B. Braun, 4657640) and sampled into 3.2 % (1:10) sodium citrate micro tubes 0.5 ml 9NC (Sarstedt, Nümbrecht, Germany). The anticoagulant to blood ratio was 1:9. Absence of clots was confirmed before centrifugation. Blood was centrifuged at 1.800 x g for 10 min at 4°C within one hour of blood collection. Plasma supernatant was collected, frozen and stored at -80°C. Coagulation assays were performed at the Clinical Laboratory of the Vetsuisse Faculty (University of Zurich, Switzerland). The activated Partial Thromboplastin Time (aPTT) and fibrinogen plasma levels were determined using a Stago Start 4 analyser (Stago Diagnostica, Asnières sur Seine, France). The aPTT tests the functionality of the contact activation pathway of blood coagulation. In brief, calcium, a platelet substitute and an activator of FXII such as kaolin was mixed with citrated plasma, and time until clot formation was recorded. C57/Bl6J are reported to have a median aPTT of 35 s with a coefficient of variation of 15% (Kopic et al., 2019). Thus, we considered a prolonged aPTT (> 40 s) as indicative of a lack of integrity of the intrinsic pathway. Three animals in the NTL and one animal in the arcAβ and arcAβ/*FXII^-/-^* groups which had a aPTT > 24 s were excluded from analysis. Fibrinogen plasma concentration was measured with the Clauss method, where diluted plasma is incubated with thrombin. The clotting time was measured and related to a standard curve of known fibrinogen concentration (Stang et al., 2013).

**Plasma FXII levels**

Mice were anesthetized with a mixture of ketamine/xylaxine (150/20 mg/kg b.w.). Blood was collected with cardiac puncture (500 uL) into ethylenediaminetetraacetic (EDTA)-coated tubes (Sarsted, Nümbrecht, Germany) and centrifuged at 1000 g for 15 min at 4°C. Plasma supernatant was aliquoted and frozen, and stored at -80°C until further use. FXII plasma levels were evaluated using a mouse FXII sandwich enzyme-linked immunosorbent assay (ELISA) (LifeSpan Biosciences Inc., Seattle, USA, Catalogue # LS-F10441) according to the manufacturer’s procedure. EDTA-plasma was diluted 1:100 000 and added to the microtiter plate already coated with an anti-mouse FXII antibody, which captured FXII present in murine plasma. Subsequently, a biotin labelled anti-mouse FXII primary antibody was added to bind to the captured FXII. Horseradish peroxidase-conjugated streptavidin secondary antibody was then incubated and bound to the primary biotinylated antibody. Following application of tetramethylbenzidine substrate quenched by HCl, the absorbance was measured at 450 nm with an Infinite® 200 Pro microplate reader (Tecan, Männedorf, Switzerland). The absorbance and known concentration of the standard curve was used to determine the FXII concentration in plasma samples based on a four-parameter logistic regression.

**Tissue factor activity assay**

Tissue factor (TF) activity was determined as previously described (Liberale et al., 2020) by colorimetric ACTICHROME® TF assay according to the manufacturer’s recommendation (American Diagnostica, Stamford, CT, USA, Catalogue # 846). Right carotid arteries were lysed (50 mmol/L Tris–HCl, 100 mmol/L NaCl, 0.1 % Triton X-100, pH 7.4), and total protein concentration was determined by Bradford protein assay according to the manufacturer’s recommendations (VWR Life Science AMRESCO, Solon, OH, USA). Arterial lysates were mixed with factor VIIa and X following the manufacturer’s instructions, which leds to the conversion of factor X to Xa; factor Xa subsequently cleaved the chromogenic substrate SPECTROZYME FXa. Optical density of cleaved SPECTROZYME FXa was determined at 490 nm by Nanodrop 2000 Spectrophotometer (Thermo Scientific, Waltham, MA, USA) and subtracted from absorbance at 405 nm. Finally, TF (pM) content was calculated, according to a standard curve and TF concentration as detected by the colorimetric assay was normalized to the total protein content of the sample and expressed as pmol/mg of total protein.

**Histological analysis/Immunohistochemistry**

Under ketamine/xylaxine (150/20 mg/kg b.w.) mice were transcardially perfused and then with ice-cold phosphate-buffered saline (PBS). Brains were removed and dissected into two hemispheres. The left hemisphere was fixed in 4% paraformaldehyde (PFA) (w/v) in phosphate buffer overnight at 4°C for while the right hemisphere was snap-frozen. Histological analyses were conducted following published procedures (Knobloch et al., 2007; Derungs et al., 2017). PFA-fixed brains were cryoprotected in 30 % sucrose solution in PBS for 72 h. Brains were cut into 35 µm thick coronal free-floating sections from position 1.7 mm to -1.3 mm (from Bregma)(Paxino and Franklin, 2003) using a microtome (Leica Jung HN40, Leica Microsystems AG, Heerbrugg, Switzerland) and kept at -20°C in an anti-freeze solution (phosphate buffer 0.5 M in MilliQ water: ethyleneglycol: glycerol = 1.3:1:1) until staining was performed. Six equally spaced sections per animal were used for immunohistochemistry.

For immunofluorescence (IF)-staining, sections were washed in PBS containing 0.1 % Tween (PBS-T), blocked for 1h in 10 % donkey serum in PBS-T for 1 h at room temperature (RT), and incubated with primary antibody in 5 % donkey serum in PBS-T overnight at 4°C. After wash in PBS-T, secondary antibody incubations were carried out for 2h at RT. 4′,6-Diamidin-2-phenylindol (DAPI) was applied as a counterstaining, sections were mounted on glass slides using Hydromount® (National Diagnostics, Atlanta, USA).

For 3,3-diaminobenzidine (DAB)-staining, brain sections were washed in Tris buffer containing 0.1 % Triton (Tris-Triton), blocked 10 min in 3 % H_2_O_2_ solution in Tris-Triton. Following blocking with 10 % donkey or goat serum at RT for 1 h, sections were incubated overnight with primary antibody in 5 % blocking serum at 4°C. After washing, secondary biotinylated antibody was applied for 2 h at RT. Then standard procedure for DAB staining was applied with the Vectastain® Elite ABC HRP kit (Vector Laboratories, Burlingame, CA, USA, Catalogue # PK-6101).

Primary and secondary antibody used for IF or DAB-staining are listed in the table below.

| Primary antibody (Manufacturer, Catalogue #, Lot #, Dilution) | Secondary antibody (Manufacturer, Catalogue #, Lot #, Dilution) |
| --- | --- |
| *Amyloid-β* | |
| Rabbit anti-amyloid fibrils OC (Millipore, Catalogue # AB2286, Lot # 3126038, 1:600) | Alexa fluor 488-conjugated Donkey Anti-Rabbit (Jackson ImmunoResearch, West Grove, PA, USA, Catalogue # 711-545-152, 1:500) |
| *Vessels – CD31 endothelial cells marker* | |
| Purified Rat Anti-Mouse CD31, Clone MEC 13.3 (BD Pharmingen, Franklin Lakes, USA, Catalogue # 553370, Lot # 7236792, 1:100) | Cy3-conjugated Donkey Anti-Rat (Jackson ImmunoResearch, Catalogue # 712-165-153, 1:500) |
| *Microglia – ionized calcium-binding adapter molecule 1 (Iba-1)* | |
| Rabbit Anti-Iba1 (Wako Chemicals, Osaka, Japan, Catalogue # 19-19741, Lot # PTH4470, 1:600) | Biotinylated Goat Anti-Rabbit (Vector Laboratories, Burlingame, CA, USA, Catalogue # 30014, Lot # ZF0326, 1:200) |
| *Astrocytes – glial fibrillary acid protein (GFAP)* | |
| Guinea pig Anti-GFAP (Origene, Rockville, USA, Catalogue # BP5082, Lot # 401081-1, 1:600) | Biotinylated-conjugated Donkey Anti-Guinea pig (Jackson ImmunoResearch, Catalogue # 706-065-148, 1:500) |

Thioflavin S staining for amyloid-β plaques was conducted according to standard protocols (Schmidt et al., 1995). The brain sections were placed into different solution and rinse in MilliQ water between each bath in the following order: (1) 0.25 % potassium permanganate 20 min, (2) a mixture of 2 % potassium disulfit and 1 % oxalic acid for 2 min, (3) 0.25 % acetic acid, (4) 0.0125 % Thioflavin S in 50% ethanol.

**Microscopy and image analysis**

Thioflavin S (IF), Iba1 (DAB) and GFAP (DAB) immunohistochemical images were visualized with a Zeiss Axio Scan.Z1 slidescanner (Carl Zeiss Microspocy, Iena, Germany) and analysed using ImageJ software (NIH, USA). High-resolution and co-localization images of Trypan Blue, Aβ and CD31 (IF) were acquired using a Leica TCS SP8 (Leica microsystem, Wetzlar, Germany) confocal microscope. CD31 (IF) images were taken with a Leica DM4000B microscope (Leica microsystem, Heerbrugg, Switzerland) using an Olympus DP71 (Olympus, Shinjuku, Tokyo, Japan) colour digital camera and newCast software (Visiopharm, Hoersholm, Denmark). Microscopy images taken were analysed using ImageJ software threshold segmentation and area measurement tools, and Ilastik (V1.3.2, European Molecular Biology Laboratory, Heidelberg, Germany). All images from one type of staining were processed under identical condition by an experimenter blinded to the genotype. The average of 5 to 6 non-adjacent sections per animal were analysed.

*Amyloid-β load (Thioflavin S)*

Quantitative image analysis of Thioflavin S staining was performed in eight ROIs. ROIs were manually delineated using the Allen Mouse Brain Atlas (Allen Institute for Brain Science., 2015; Lein et al., 2007) for anatomical references: Cortex (1.045 mm to -1.855 mm), Hippocampus (-1.455 mm to -1.855 mm), Caudate Putamen (1.045 mm to -1.055 mm), Lateral Septal Complex (LSx) (1.045 mm to 0.020 mm), Nucleus Accumbens (NAcc) (1.045 mm to 0.745 mm), Pallidum/Olfactory tubercle (1.045 mm to 0.145 mm), Hypothalamus (0.145 mm to -1.855 mm), Thalamus (-0.655 mm to -1.855 mm), Pallidum (0.020 mm to -1.055 mm), Striatum-like amygdalar nuclei (sAMY) (-0.48 mm to -1.855 mm) (all coordinates are given from Bregma according to the Allen Mouse Brain Atlas (2015). Not all the coronal sections included all brain areas selected for analysis (such as NAcc and hippocampus). For the hippocampus and thalamus, only ROI superior to 1800 000 pixels were considered. The area of each ROI and labelled object was measured in pixel with ImageJ Otsu segmentation and area measurement tools. For each animal, as the same ROI appeared on 2 to 6 subsequent sections, the plaque load was calculated as the mean fraction of labelled object weighted with the total number of pixel in that ROI (Σ pixel labelled object ROI1/Σ pixel ROI1 × 100).

*Microglia (Iba-1) and astrocytes (GFAP) coverage*

Quantitative image analysis of Iba-1 and GFAP was performed in the cortex and hippocampus according to the coordinates mentioned above. The percentage of area covered by Iba-1/GFAP was quantified using ImageJ threshold segmentation and area measurement tools. The quantification resulted from the average of 5 to 6 sections per animal. Not all animals presented the hippocampus on the brain sections, explaining the smaller number of samples used for the ROI hippocampus.

*Trypan Blue extravasation*

Trypan Blue was imaged with an excitation and emission wavelengths at 642 and 660 nm respectively. For each animal, 3 to 4 pictures in the isocortex were taken per brain section on five sections. Trypan extravasation was quantified as the percentage of area covered by Trypan Blue over total area, using ImageJ software. The same procedure was used to quantify the area covered by Aβ fibrils OC staining. Trypan Blue extravasation related to Aβ plaques was calculated as the percentage of Trypan Blue area relative to Aβ coverage. When the capillary pattern was seen on several confocal picture, the perfusion was considered as not complete and the animal was excluded from the analysis, as it was not possible to distinguish between Trypan Blue extravasation and vessels that were not well perfused. Two mice in the NTL and *FXII^-/-^* groups and one mouse in the arcAβ/*FXII^-/-^* group were excluded for this reason.

*Vessel density (CD31)*

For quantification of CD31 vessel staining, the image was first segmented with Ilastik software and then the vessel density was calculated as the percentage of area covered by CD31 positive area with ImageJ area measurement tool.

**Statistics**

All statistical analysis was performed using GraphPad Prism software (V7.00, San Diego, CA, USA). Normality and variance homogeneity was tested with Kolmogorov–Smirnov test and variance homogeneity was assessed with Fisher test for comparison between two groups and Bartlett’s test for comparison between more than two groups. As several data sets did not meet the requirement of normality distribution and homoscedasticity, or presented small sample size, statistical analyses were conducted using non-parametric tests as a matter of consistency, except for the statistical analysis of the latency to success in the Barnes maze. For comparison between two groups, Wilcoxon test was used. For comparison between several groups, Kruskal-Wallis test followed by *post hoc* Dunn’s correction for multiple comparison was applied. The latency to success in the learning phase of the Barnes maze was analysed by 4 × 4 (genotype × days) two-way analysis of variance (ANOVA) with repeated measures (RM). Tukey’s post hoc test correction for multiple comparison was performed following ANOVA for analysis of the simple effects. A *p*-value < 0.05 was considered as statistically significant. To go beyond the difference determined by statistical tests and give quantitative information about the difference between two groups when statistical significance was reached and when the assumption of normality was reasonable, we calculated the effect size using Cohen’s d (Cohen, 1988). We interpreted the intensity of Cohen’s d effect size according to the scale published (Sawilowsky, 2009) and our knowledge of the variable assessed.

**References**

Allen Institute for Brain Science. (2015). Allen Mouse Brain Atlas. Available from: <http://mouse.brain-map.org/>.

Brooks, S.P., et al. 2009. Tests to assess motor phenotype in mice: a user's guide. *Nat Rev Neurosci* **10**, 519-529.

Cohen, J., 1988. Statistical Power Analysis for the Behavioral Sciences. NY: Routledge Academic.

Derungs, R., et al., 2017. Genetic ablation of the p66(Shc) adaptor protein reverses cognitive deficits and improves mitochondrial function in an APP transgenic mouse model of Alzheimer's disease. Mol Psychiatry 22, 605-614.

Gawel, K., et al., 2019. Assessment of spatial learning and memory in the Barnes maze task in rodents-methodological consideration. Naunyn-Schmiedeberg's archives of pharmacology 392, 1-18.

Harrison, F.E., et al., 2006. Spatial and nonspatial escape strategies in the Barnes maze. Learn Mem 13, 809-819.

Hurst, J.L., et al., 2010. Taming anxiety in laboratory mice. Nat Methods 7, 825-826.

Illouz, T., et al., 2016. Unbiased classification of spatial strategies in the Barnes maze. Bioinformatics 32, 3314-3320.

Klohs, J.*,* et al., 2011. Detection of cerebral microbleeds with quantitative susceptibility mapping in the ArcAbeta mouse model of cerebral amyloidosis. J Cereb Blood Flow Metab 31, 2282-2292.

Knobloch, M., et al., 2007. Intracellular Abeta and cognitive deficits precede beta-amyloid deposition in transgenic arcAbeta mice. Neurobiol Aging 28, 1297-1306.

Kopić, A.*,* et al., 2019. Coagulation phenotype of wild-type mice on different genetic backgrounds. Lab Anim 53, 43-52.

Kulic, L.*,* et al., 2012. Early accumulation of intracellular fibrillar oligomers and late congophilic amyloid angiopathy in mice expressing the Osaka intra-Abeta APP mutation. Transl Psychiatry 2, e183.

Lein, E.S.*,* et al., 2007. Genome-wide atlas of gene expression in the adult mouse brain. Nature 445, 168-176.

Liberale, L.*,* et al., 2019. Interleukin-1beta Mediates Arterial Thrombus Formation via NET-Associated Tissue Factor. J Clin Med 8(12):2072.

Liberale, L., et al., 2020. Sirtuin 5 promotes arterial thrombosis by blunting the fibrinolytic system. Cardiovasc Res, 117(10):2275-2288.

Merlini, M., et al., 2011. Vascular beta-amyloid and early astrocyte alterations impair cerebrovascular function and cerebral metabolism in transgenic arcAbeta mice. Acta Neuropathol 122, 293-311.

Ni, R., et al., 2020. SWI and phase imaging reveal intracranial calcifications in the P301L mouse model of human tauopathy. MAGMA 33, 769-781.

Pauer H.U., et al., 2004. Targeted deletion of murine coagulation factor XII gene-a model for contact phase activation in vivo. Thromb Haemost 92, 503-508.

Paxinos G, Franklin K, 2003. The Mouse Brain in Stereotaxic Coordinates. Academic Press.

Persson, L., et al., 1976. Extravasation, spread and cellular uptake of Evans blue-labelled albumin around a reproducible small stab-wound in the rat brain. Acta Neuropathol 34, 125-136.

Pompl, P.N., et al., 1999. Adaptation of the circular platform spatial memory task for mice: use in detecting cognitive impairment in the APP(SW) transgenic mouse model for Alzheimer's disease. J Neurosci Methods 87, 87-95.

Rosenfeld, C.S., et al., 2014. Barnes maze testing strategies with small and large rodent models. *J Vis Exp*, e51194.

Sawilowsky, S.S., 2009. New Effect Size Rules of Thumb. Journal of Modern Applied Statistical Methods 8, 597-599.

Schmidt, M.L., et al., 1995. Chemical and immunological heterogeneity of fibrillar amyloid in plaques of Alzheimer's disease and Down's syndrome brains revealed by confocal microscopy. Am J Pathol 147, 503-515.

Stang, L.J., et al., 2013. Fibrinogen. Methods Mol Biol 992, 181-192.

Zamolodchikov, D., et al., 2016. The Alzheimer's disease peptide beta-amyloid promotes thrombin generation through activation of coagulation factor XII. J Thromb Haemost. 14**,** 995-1007.
