# Supplementary material for: Coagulation factor XII deficiency alleviates vascular dysfunction and cognitive impairment in a mouse model of cerebral β-amyloidosis and cerebral amyloid angiopathy
Source: Aging Brain. 2026 Aug 25;10:100167. doi: 10.1016/j.nbas.2026.100167 (PMC13543942; doi:10.1016/j.nbas.2026.100167)
Supplement: Supplementary file 2 [file mmc2.docx]

**Supplementary Figures**

**Supplementary Figure 1. Tissue factor activity.** Tissue factor activity measured in the carotid artery was not significant between 15-month-old NTL, arcAβ and arcAβ/*FXII^-/-^* groups. Data presented as mean ± SD. NTL (n=5), arcAβ (n=8) and arcAβ/*FXII^-/-^* (n=5); Kruskal-Wallis test with *post hoc* Dunn’s correction for multiple comparisons.

**Supplementary Figure 2. Vessel density.** (**a**) Representative cortical CD31 vessel staining of NTL, *FXII^-/-^*, arcAβ and arcAβ/*FXII^-/-^* groups. The background was subtracted equally and contrast enhanced in all pictures presented in the figure. (**b**) The segmentation of the microscopy image above shows a clear representation of the vessel pattern. Scale bar = 400 μm. (**c**) Quantification shows no difference in vessel density between all experimental groups. Data presented as mean ± SD. NTL (n=8), *FXII^-/-^* (n=8), arcAβ (n=8) and arcAβ/*FXII^-/-^* (n=8); Kruskal-Wallis test, non-significant.

**Supplementary Figure 3. Aβ neuropathology.** (**a**) Quantification of Thioflavin S staining in different cerebral area as shown in (**b**) cortex (1), hippocampus (2), thalamus (3), hypothalamus (4), and striatum-like amygdalar nuclei (5). No difference in Aβ load was observed between arcAβ and arcAβ/*FXII^-/-^* mice in these regions. Data presented as mean ± SD. arcAβ (n=15) and arcAβ/*FXII^-/-^* (n=14); Wilcoxon test.

**Supplementary Figure 4**. **Cognitive performance in the Barnes maze of 15-month-old mice**. (**a**) Latency to success (i.e. latency to enter the target box) was measured in all trials in the learning phase. (**b**) In the probe trial, latency to first entry the target hole after the start of the test was recorded, and showed no difference among groups in the Kruskal-Wallis test. Nevertheless, a Wilcoxon test, comparing the two groups arcAβ and NTL, showed significant increased latency in arcAβ mice compared to NTL mice (**p < 0.001). Data presented as mean ± SD. NTL (n=17), *FXII^-/-^* (n=26), arcAβ (n=13) and arcAβ/*FXII^-/-^* (n=10).

**Supplementary Figure 5. Examples of search strategy during the learning phase of the Barnes maze.** The search pattern were classified into six search strategies: random (the mouse does not show ability to locate the target hole and moves randomly on the platform), serial (the mouse visits several adjacent holes in a serial manner), focused search (the mouse scans the location around the target hole), long correction (the mouse moves to a distal position from the target hole and makes a major correction re-directing its trajectory towards the target hole), corrected (the mouse trajectory does a minor correction toward the target hole), and direct (the mouse moves directly toward the target hole and enter the target box). The three search strategy serial, focused search, long correction were grouped in the serial search category (blue). The most highly spatial strategy corrected and direct were grouped in the category spatial (in green). The search strategy random was not grouped with any other search strategy was considered in the group random (in purple).
